# Assessing Human Ribosomal DNA Variation and Its Association With Phenotypic Outcomes

**DOI:** 10.1002/bies.202400232

**Published:** 2025-01-20

**Authors:** Francisco Rodriguez‐Algarra, Elliott Whittaker, Sandra del Castillo del Rio, Vardhman K. Rakyan

**Affiliations:** ^1^ The Blizard Institute School of Medicine and Dentistry Queen Mary University of London London UK

## Abstract

Although genome‐scale analyses have provided insights into the connection between genetic variability and complex human phenotypes, much trait variation is still not fully understood. Genetic variation within repetitive elements, such as the multi‐copy, multi‐locus ribosomal DNA (rDNA), has emerged as a potential contributor to trait variation. Whereas rDNA was long believed to be largely uniform within a species, recent studies have revealed substantial variability in the locus, both within and across individuals. This variation, which takes the form of copy number, structural arrangement, and sequence differences, has been found to be associated with human phenotypes. This review summarizes what is currently known about human rDNA variation, its causes, and its association with phenotypic outcomes, highlighting the technical challenges the field faces and the solutions proposed to address them. Finally, we suggest experimental approaches that can help clarify the elusive mechanisms underlying the phenotypic consequences of rDNA variation.

## Graphical abstract

Genetic variation within the multi‐copy, multi‐locus ribosomal DNA (rDNA) can contribute to human complex trait variability. This variation takes the form of copy number, structural arrangement, and sequence differences. This review summarizes what is currently known about human rDNA variation, its causes, and its association with phenotypic outcomes.

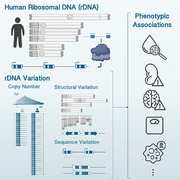

## 1 Introduction

The widespread adoption of genome‐scale analyses has expanded our understanding of how genetic variation impacts complex human traits. In many cases, however, much of the trait variation remains unexplained. Large‐scale cohorts often identify rare genetic variants as key contributors for yet‐unexplained trait variance, but variation in repetitive genomic regions seldom considered in such studies can also help explain differences in phenotypic outcomes. These include the multi‐copy, multi‐locus ribosomal DNA (rDNA), a fundamental feature encoding for ribosomal RNAs (rRNAs) throughout the tree of life [1]. Recent studies have employed large‐scale whole genome sequencing (WGS) cohorts [2, 3, 4] and long‐read technologies [5, 6, 7, 8, 9] to better characterize human rDNA variation and start elucidating its phenotypic consequences. We here summarize the current knowledge in human rDNA variation and its association with phenotypic outcomes, largely focusing on the 47S rDNA. We also propose approaches to uncover the mechanisms underlying the causal impacts of rDNA variation.

## 2 The Human rDNA

**FIGURE 1:**
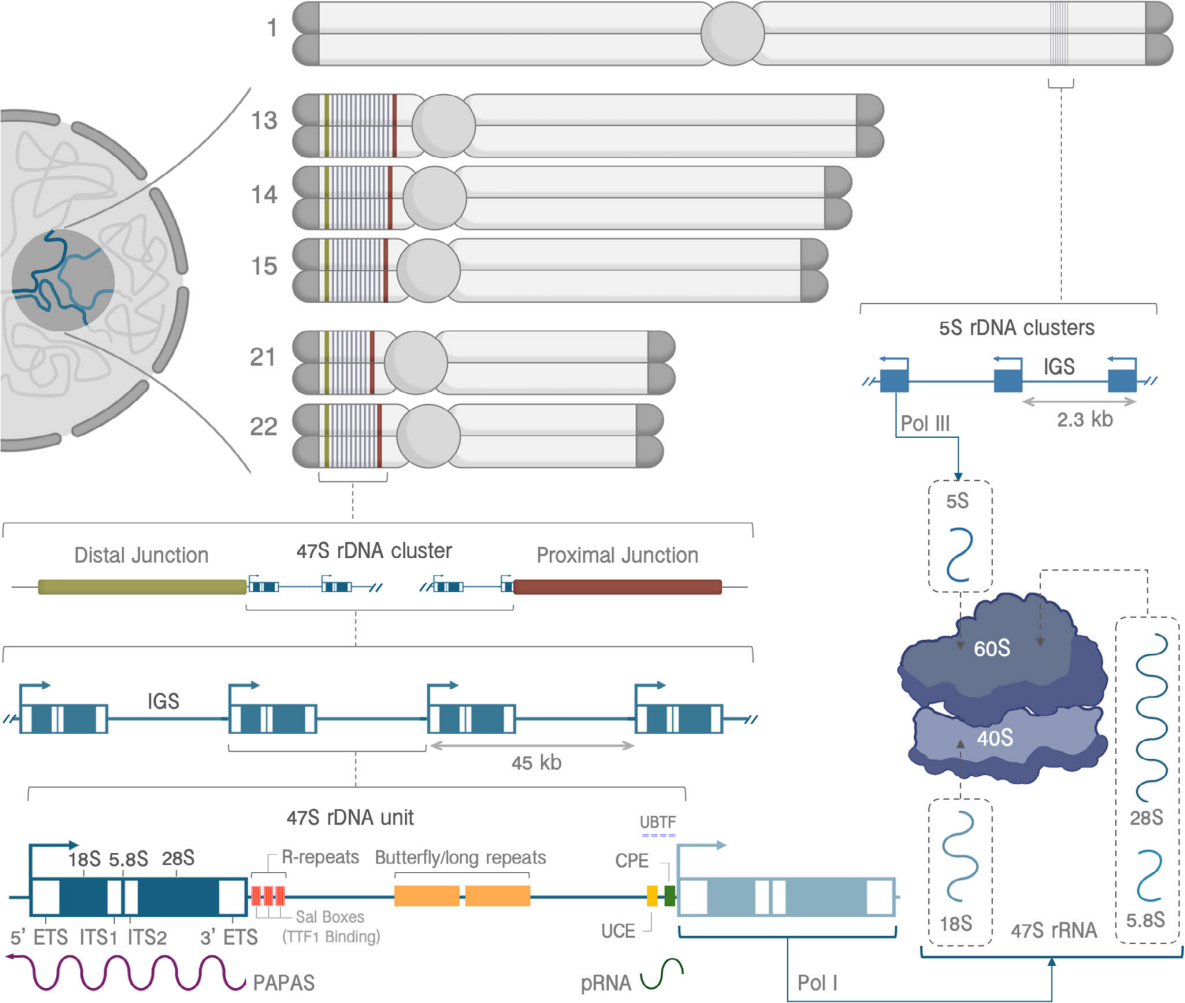
Schematic representation of the human Ribosomal DNA loci. In humans, the 47S rDNA clusters, which gather in the nucleolus, are present across the short arms of the five acrocentric chromosomes as tandem repeats flanked by the Distal Junction and Proximal Junction sequences. Each unit is around 45 kb long and contains a coding region—including the 18S, 5.8S, and 28S rDNAs as well as internal and external transcribed spacers—and an intergenic spacer (IGS). The IGS harbors several repeated sequences, such as the R‐repeats, whose “Sal boxes” bind Transcription Termination Factor 1 (TTF1) and thus mark the end of the 47S rRNA transcript, and the Butterfly/Long repeats—each consisting of, as the name suggests, a Long and a Butterfly repeat, separated by a CT‐rich region. Transcripts of non‐coding RNAs, such as PAPAs and pRNA, cover at least partly the promoter region of the rDNA unit and regulate rRNA expression. The promoter itself is located just upstream of the transcription start site and contains core (CPE) and upstream (UCE) elements. The binding of UBTF in that region recruits SL1, essential for RNA Polymerase I (Pol I) transcription of pre‐rRNA, which is later processed to produce the mature 18S rRNA (incorporated into the 40S small ribosomal subunit), and 5.8S and 28S rRNAs (incorporated into the 60S large ribosomal subunit). The 5S rRNA, the final RNA component of the 60S, is transcribed by Pol III from clusters of 2.3 kb tandem repeats located in chromosome 1.

Few processes are as fundamental to life as protein synthesis. Ribosomes, the molecular machines in charge of this process, employ around 80 ribosomal proteins (RPs) and four RNAs [10], which requires high rRNA transcription [11]. The corresponding genomic loci, the rDNA, typically contain over 100 copies per eukaryotic genome [12]. In humans, the 47S and 5S rDNA operons are encoded in separate chromosomes—the 2.3 kb 5S units appearing as tandem arrays on chromosome 1 [13, 14], and the approximately 45 kb 47S rDNAs (hereinafter referred to simply as “rDNA”) on the short arms of the acrocentric chromosomes (13, 14, 15, 21, and 22) [15] (Figure 1). These clusters form the Nucleolus Organizing Regions (NORs), flanked by the Proximal Junction (PJ)—showing some diversity in sequence and location—and the Distal Junction (DJ)—virtually invariant in sequence and placement [6, 16, 17]. NORs from different chromosomes often join to form human nucleoli [18].

A typical human rDNA unit consists of the 13 kb‐long 47S rRNA operon and the 30 kb‐long intergenic spacer (IGS) [19, 20]. The former includes the 18S (1.8 kb), 5.8S (0.15 kb), and 28S (5kb) separated by “internal transcribed spacers”—ITS1 and ITS2—and flanked by “external transcribed spacers”—5′ ETS and 3′ ETS [21]. Whereas the coding rDNA regions are highly conserved among eukaryotes, with an estimated similarity between human and budding yeast ranging from 50% to 75% [22], the spacers are largely variable both in length and sequence. The size of the IGS appears to have been greatly increased in a mammalian ancestor, with all known non‐mammalian animals having less than 20 kb [23]. Across primates, the IGS varies in length, between 25 and 30 kb, and content, such as the presence of pseudogenes [24, 25]. Even within humans, a consensus IGS sequence has proven challenging to determine, with recent increases in the overall length of the reference unit, from 43 kb [20] to 44 kb [25] and 45 kb [26], largely due to a 2 kb tandem repeat within the IGS. This latest version, named KY962518.1, largely coincides with one of the rDNA clusters from the human Telomere‐to‐Telomere (T2T) assembly [6] and has been widely adopted in the community [27].

The IGS also harbors transposable—mainly SINE/Alu—and other repetitive elements. Whereas the function of some of those sequences remains obscure, such as the conserved 4.5 kb Butterfly/Long repeat halfway through the IGS [20, 25, 28], others have essential roles. Downstream of the 3′ ETS lie the “R repeats”—three tandem copies of approximately 690 bp each in KY962518.1 [5, 26]. The T2T assembly, however, reports these as copy‐number polymorphic [6], consistent with previous studies [29]. They contain the 18 bp Sal box sequence associated with transcription termination factor TTF1 [30, 31], which marks the end of the 47S rRNA transcript by arresting RNA Polymerase I (Pol I), acting as a Replication Fork Barrier [32]. Every other transcript in the genome, including the 5S rRNA, is transcribed by either Pol II or Pol III.

The rDNA gene promoter, located upstream of the transcription start site (TSS) at the 5' end of the 5' ETS, is approximately 150 bp long and comprises a core element (CPE) and an upstream control element (UCE) [33, 34]. A TTF1 binding site just upstream from the promoter may protect the gene promoter from the activity of RNA polymerases and recruit transcription factors [35, 36, 37]. In particular, the binding of Upstream Binding Factor (UBF, sometimes also called UBTF) in the region recruits SL1, thus regulating rRNA transcription activity [38, 39], with only around half of the rDNA units being transcribed at any given time [40]. In other organisms, an enhancer element has been identified upstream of the UCE [41, 42], but its precise location in humans remains unknown [43, 44].

Beyond the 47S rRNA, other transcripts also arise from the rDNA repeats, expressed at substantially lower levels [45 ^–^ 48]. One of these sequences, commonly referred to as pRNA, spans the 3′ end of the IGS and part of the 5′ ETS and is involved in rDNA silencing in *trans* [49, 50]. An even larger transcript called “promoter and pre‐rRNA anti‐sense” (PAPAS) also contributes to repressing rRNA synthesis through a separate mechanism. Its >10 kb product is transcribed by Pol II in the anti‐sense direction, spanning part of the coding region, the promoter, and the putative enhancer [51]. The 10 kb Pyrimidine‐rich Non‐Coding Transcript (PNCTR) is transcribed by Pol I from a CT‐rich region within the IGS, and appears to be expressed at higher levels in cancer cells than in normal cells [52]. Under stress, this and other transcripts derived from the IGS region may contribute to “nucleolar detention,” identifying and arresting specific proteins within the nucleolus [53 ^–^ 55]. A more detailed discussion about the various transcripts that have been previously identified in the region and their function can be found elsewhere [56].

## 3 Human rDNA Genetic Variation

**FIGURE 2:**
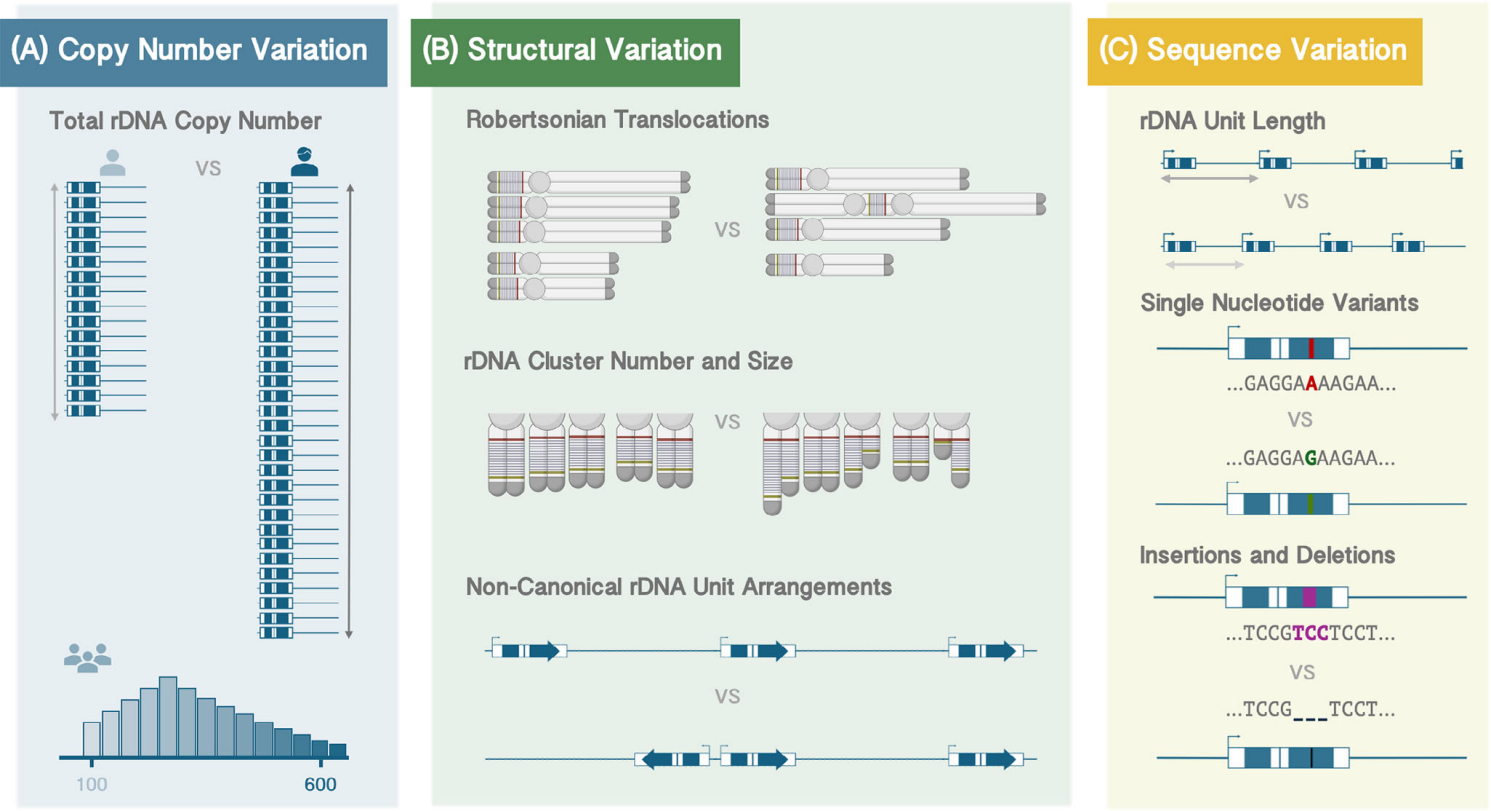
Types of human rDNA variation. We divide the distinct types of rDNA variation in the human genome into three categories. (A) First, cells in each individual harbor a specific total number of rDNA copies across the five acrocentric chromosomes, usually ranging between 200 and 600 copies throughout the human population. (B) Structural variation refers to any change in the arrangement of the rDNA clusters and entire units. This includes chromosomal abnormalities, such as Robertsonian Translocations, where two acrocentric chromosomes fuse through their short arms and thus full rDNA clusters are lost. Even across healthy individuals, the size of rDNA clusters can display different chromosomal distributions. Some studies also report the presence of units with reverse orientation and in palindromic structures. (C) Finally, both inter‐ and intra‐genomic sequence variants exist. rDNA units of varied length, largely due to number‐variable repetitive elements within the IGS, have been reported, and both Single Nucleotide Variants (SNVs) and Insertions and Deletions (INDELs) exist both inside and outside of the rRNA‐coding regions.

Although intragenomic variation within the human rDNA has been known for almost 50 years [57], the long‐accepted view in the field was that, given how essential rRNAs are, rDNA repeats undergo “concerted evolution” [58]—rDNA units may differ across species but are heavily homogenized within each species. Estimates of the level of rDNA variability in some fungi species appeared to confirm this hypothesis [59]. Nevertheless, the constant rRNA transcription [60] and the repetitive nature of the loci can make rDNA one of the most unstable regions in the genome [61]. In addition, multiple regions within the rDNA unit display the hallmarks of recombination hotspots [28, 62], which can occur between distant units [63]. Chromosome exchanges in the rDNA during meiosis can exceed 10% per generation and cluster, enabling them to quickly restructure [62]. This may introduce variability through deletions, duplications, or even the complete elimination of coding sequences [26], aside from alteration in the total number of copies [64, 65]. In recent years, the availability of larger and better‐quality sequencing datasets has revealed that rDNA variation is ubiquitous, beyond what concerted evolution would predict [66, 67], and appears at multiple levels, including the number of copies each individual harbors, the arrangement of those copies, and the nucleotide sequence that forms each copy [1, 68] (Figure 2).

### 3.1 Copy Number Variation

Across species, the total number of rDNA copies of an organism correlates with the species’ genome size [69]. In humans, the rDNA copy number (CN) is estimated to be between 200 and 600 [70] (Figure 2A), although some studies reported estimates from healthy individuals as high as 1500 copies, and as low as 9 or 14 [2, 71, 72]. This low end seems to contradict evidence from other species since organisms with excessively low rDNA CN cannot support ribosome biogenesis and suffer serious developmental complications [73, 74, 75]. Further analyses, however, suggest these extreme values may have been technical artifacts due to low‐coverage data [76], highlighting that rDNA copy number estimation remains challenging.

Various electrophoretic [62] and PCR‐based [77 ^–^ 79] methods have been used to estimate rDNA CN. More recent analyses, however, calculate rDNA CN from short‐read WGS datasets by comparing mapping depth between the rDNA and the rest of the genome. WGS data, however, can suffer from library preparation biases and batch effects [76, 80], and their coverage distributions are sensitive to factors such as the GC content of the underlying sequence [3]. Some have thus proposed statistical methods to mitigate their impact [2, 81]. Nevertheless, comparisons between ddPCR and sequencing‐based rDNA CN estimates reveal these two orthogonal methods highly correlate [7]. Moreover, carefully selecting genomic regions may permit reliable rDNA CN estimates from pre‐aligned libraries [4], even though rDNA units are not explicitly included in standard human genome assemblies [27]. These estimates, however, should be treated cautiously, and perhaps only interpreted as relative comparisons within a cohort and not necessarily reflective of the actual absolute number of copies per individual. T2T assemblies from ultra‐long‐read WGS data may precisely quantify rDNA CN [6], but their current cost makes them unfeasible to use at scale.

Regardless of the technical challenges in its estimation, inter‐individual rDNA CN variation is undeniable and appears to differ across ancestries [2, 4]. The repetitive nature of the rDNA accentuates its meiotic instability, which in turn promotes changes in the number of copies across generations [82, 83], despite being a heritable trait [84]. Given this instability, however, it is not implausible that intra‐individual variation might also exist. Although intense rDNA amplification has been observed in oocytes of various non‐mammalian species [85, 86], to date no credible report has shown rDNA CN variation across somatic tissues of a single individual [73, 77]. Moreover, familial comparisons of rDNA CN from whole blood samples in humans display similar heritability to the rest of the genome, despite their likely diverse blood composition at sampling time [4]. This further suggests rDNA CN is somatically stable in healthy tissues.

It is reasonable to wonder how cells balance meiotic instability, necessary for inter‐individual variation, and CN maintenance through mitosis. To this end, cells employ a refined amplification mechanism to compensate for CN loss based on recombination among repeats through DNA damage and posterior repair [61]. Nevertheless, considerably more is known about these processes in lower organisms than in mammals [22]. In yeast, for example, unequal sister chromatid exchange is suppressed in the clusters during mitosis [87, 88], and extrachromosomal circles can be reinserted into the genome to recover lost copies [89 ^–^ 91], allowing them to adjust their number of rDNA copies in response to nutrient availability [92]. Flies, on the other hand, can recover lost rDNA copies over a few generations through unequal chromatid exchange [93 ^–^ 97]. Although it is unknown whether human cells rely on similar mechanisms, the similarity between contiguous rDNA units [5, 6] suggests that might be the case [59].

Some authors have argued that these rDNA CN maintenance mechanisms coordinate the number of copies of 5S and 47S rDNA in both humans and mice, even if they are located in different chromosomes [71, 72]. Similar to the extreme CN values mentioned above, however, analyses of higher‐quality WGS datasets found no substantial correlation between 5S and 47S rDNA CN in humans [76]. Comparisons across multiple species also showed no significant correlation between 5S and 47S rDNA CN [98].

### 3.2 Structural Variation

Given the total number of rDNA units in a genome, these may be arranged in a multitude of ways. By “structural variation” here we mean large‐scale changes spanning one or more units, either at the level of full clusters, such as their number or chromosomal location, or full individual units, such as their orientation (Figure 2B). Variation occurring within rDNA units—even large insertions, deletions, or duplications—falls under the category of “sequence variation” below.

Across species, rDNA clusters differ in their location within the chromosomes, being either subtelomeric, pericentromeric, or interstitial, such as the 5S cluster in human chromosome 1 [98, 99]. In humans with standard karyotype, the 47S rDNA clusters are located on the short arms of the five acrocentric chromosomes, but some studies suggest potential variation in which and how many human chromosomes harbor rDNA. In particular, Fluorescence In Situ Hybridization (FISH) on metaphase chromosome spreads suggested that, out of the 10 potential rDNA clusters on healthy human donors, most individuals lack at least one cluster [100], likely due to the high rate of meiotic recombination events in the locus [62, 100]. The size of each cluster, both within and across individuals, also appears highly variable, with estimates ranging from a single rDNA unit to over 130 units per cluster [62].

Karyotype abnormalities involving the acrocentric chromosomes affect the abundance of rDNA clusters. This is the case in individuals with Down's (trisomy of chromosome 21) [101, 102] or Patau's (trisomy of chromosome 13) [103] syndrome, leading to additional rDNA clusters [84, 104]. Other chromosomal abnormalities lead to the loss of rDNA clusters, such as in Robertsonian Translocations (ROBs), which arise from end‐to‐end chromosome fusions [105]. They are the most common chromosomal changes in mammals [106]. In mice, frequent ROBs lead to high variability in karyotype [107], contributing to speciation through reproductive isolation [108]. In humans, ROBs arise from two acrocentric chromosomes fusing their short arms, and thus losing copies of the corresponding sequences, including rDNA clusters. Large‐scale cohorts suggest that 1 of every 800 humans carry ROBs [109 ^–^ 111], with around half being inherited and the other half appearing *de novo* [112]. These translocations often fuse two maternal chromosomes, which suggests they happen during female meiosis [113, 114]. Not all fusions are equally likely, with over 90% of the observed ROBs occurring between distinct chromosomes and retaining two centromeres [115]. The vast majority of these involve chromosome 14 with either 13 or, less frequently, 21, and are speculated to originate from a homologous repetitive sequence near the rDNA clusters on chromosomes 13 and 21 that is inverted in chromosome 14 [116]. The T2T human assembly indeed identified a candidate segmental duplication fitting this description, named Pseudohomolog Region or PHR [6, 117]. Sequence homology of this kind across non‐homologous chromosomes, primarily involving repetitive DNA, is believed to be essential to form ROBs, alongside meiotic recombination and the three‐dimensional proximity of these homologous sequences [118]. The gathering of rDNA clusters into nucleoli may thus facilitate these events.

Within the clusters, the canonical view is that all rDNA units are arranged in a head‐to‐tail manner, facing the same orientation. Molecular combing analyses, however, observed around a third of the rDNA units displaying non‐canonical arrangements, namely palindromic and inverted structures, in various human cell types [119]. Later, transformation‐associated recombination (TAR) cloning experiments appeared to confirm these non‐canonical rDNA structures [26]. More recent studies relying on long‐read sequencing data, however, do not observe these structures beyond potential artifactual chimeric reads [5 ^–^ 7]. The reason behind such distinct observations is still unclear.

### 3.3 Sequence Variation

The final category of rDNA variation occurs within individual rDNA units at the DNA basepair sequence itself, including single‐nucleotide polymorphisms (SNPs) as well as insertions and deletions (INDELs) within individual units (Figure 2C). rDNA sequence variants appear across the tree of life, shaping the structure of rRNAs throughout evolution [120, 121]. Intragenomic rDNA variation has been identified in both prokaryotes [122, 123] and eukaryotes, from yeast [124, 125] and plants [126, 127] to a variety of animals: water fleas [128], nematodes [129, 130], fish [131], and mammals, particularly mice [132 ^–^ 134]. In humans, rDNA sequence variation is observed both across and within individuals [2, 3, 8, 135], at a rate at least comparable to variants elsewhere in the genome [26], to the extent that they may enable distinguishing between NORs [6, 136, 137].

This widespread evidence for intraspecies and, especially, intragenomic rDNA variation contrasts with the traditional view that rDNA units within eukaryotic genomes are virtually homogeneous yet distinct across related species, suggesting that their evolution is concerted [58, 59, 138, 139]. Computational models from half a century ago showed this homogenization process can be explained through unequal crossovers between clusters, leading to any mutation in the rDNA being erased after a few generations [140]. Nevertheless, unit‐to‐unit sequence variation exists, with some variants not only being conserved across individuals of the same species but also across evolution, such as through primates [141, 142] and even between humans and mice [2]. Concerted evolution cannot explain the existence of such conserved variants, suggesting selection pressures prevent their removal through homogenization [141, 142]. Ignoring rDNA variation can lead to invalid phylogenetic relationships [143] and overestimation of species diversity [129].

Factors such as the size of the clusters or their location within the chromosomes may influence how efficient homogenization processes are [67]. The degree of homogenization of individual rDNA units may also depend on their location within their cluster. In particular, copies at cluster edges display less homogenization, resulting in the pseudogenization and inactivation of approximately 5% of rDNA units in humans through the accumulation of mutations [144]. *De novo* variants have also been identified across generations in flies [145] and tissues in humans [77, 146]. Evidence from frogs suggests that extrachromosomal rDNA in primordial cells might also give rise to new variants through recombination with rDNA clusters immediately before meiosis, which then might be subjected to selection and spread [147, 148]. Individual units may thus be susceptible to degradation and functional impairment, suggesting homogenization processes mainly work as correction mechanisms to compensate for such instability [67].

Homogeneity across rDNA units may have been overestimated in the past due to methodologies only capable of detecting variants common both within and across individuals, such as a G>A SNV located 60 bp downstream of the 28S 5′ end in virtually all humans [149]. Analyses based on WGS data, on the other hand, reveal a wider spectrum of low‐frequency variants [2, 3, 8]. These approaches, however, are not exempt from potential pitfalls, including PCR amplification biases and depletion of GC‐rich sequences, which may cause both false negative and false positive variant calls [8]. This may be particularly relevant in the rDNA, where the GC content of some regions reaches 80% [150] and might in itself be a driver of variability [151]. Moreover, excessively low read coverage [2, 76, 80] or the omission of a subset of alignments [3] known to harbor rDNA reads [4], may have led to inconsistent results across studies. The choice of variant calling software, even when considering tools such as Mutect2 [152] and Lofreq [153] that avoid ploidy assumptions, also affects the breadth and type of variants detected [8]. Finally, although short‐read Illumina reads are widely believed to be highly accurate, unpublished data from our laboratory comparing variant calls from monozygotic twins suggests that low‐frequency apparent T>G (and equivalent A>C) changes might be artifactual. Novel methodological approaches, such as Reference Gap Alignment (RGA), and the analysis of both short‐ and long‐read data at both DNA and RNA levels, have been recently employed in an attempt to mitigate some of these pitfalls [8].

Regardless of the specific methodology, sequencing‐based studies report abundant intragenomic sequence variation throughout the human rDNA unit [2, 3, 8]. Unlike variants elsewhere in the genome, the hundreds of rDNA repeats enable variation at a finer scale than the conventional homozygous/heterozygous binary. For example, one individual with 400 rDNA copies in their genome may have 300 of these containing a G (the reference allele) at position 7980, whereas the rest contain an A instead. In this case, we would say that the individual has an intragenomic frequency of 0.25 (25%) for variant 7980:G>A. The most recently published study [8] reports over 1500 of these variant positions (around 33 per kb) when analyzing short‐read data, with close to a 75/25 split in SNVs versus INDELS, a rate similar to previous reports [3]. When employing their novel RGA method on long‐read PacBio Hi‐Fi data, however, the proportion of INDELs they identified skyrocketed to around 95%, mostly within GC‐rich sequences [8]. This might suggest that short‐read sequencing data misses this variation due to its inherent biases, but independent validation is still necessary to determine how susceptible RGA is to false positives. Moreover, most detected variants appeared at intragenomic frequencies below the previously reported proportion of 5% rDNA pseudogenes [144]. Further work remains for the community to establish best practices for validating intragenomic variant calls.

Although variants appear throughout the rDNA unit, they are not evenly distributed. The coding region is substantially more conserved than the rest [2, 3, 8, 154], with the IGS harboring most variants [26, 155], particularly as short tandem repeats [3]. Nevertheless, intragenomic variants in the coding subunits have been known for decades [135, 149, 156 ^–^ 158], with some having been observed in actively translating ribosomes [7, 8]. Even across the coding subunits, the distribution of variants is far from uniform. 28S variation is more common than in 18S or 5.8S [2, 8], largely falling within subdomains called expansion segments [2, 3, 8, 154]. These subdomains, which cover almost half of the 28S and over a third of the 18S, are the most divergent across species and cause the large rRNA size difference between prokaryotes and eukaryotes [159, 160]. They tend to be GC‐rich and of lower complexity than the rest [161], display signatures of purifying selection [162], and appear to match mRNA sequences [163]. Their specific function, however, remains obscure [164, 165]. Variation in the expansion segments appears to alter the 3D structure of the ribosomes, so researchers speculate these variants may fine‐tune mRNA translation [8]. Furthermore, 28S variants both in and out of expansion segments show some low level of linkage disequilibrium, and haplotypes defined from a subset of those positions appear to cluster at specific chromosomes in the T2T assembly, suggesting coding rDNA subtypes may exist in humans and be chromosome‐specific [8]. Nevertheless, these putative haplotypes still contain far more unexplained variance than the subtypes previously identified in inbred mice [7]. Refinement of human rDNA variant calls might thus still be necessary.

Variation in the spacer regions does not directly affect the composition of mature ribosomes, but the presence (or absence) of specific sequences may lead to substantial differences in rDNA functional status [166]. Moreover, the length of each full rDNA unit can differ [65], potentially altering rRNA synthesis if promoter or enhancer regions get affected [167]. In humans, multiple studies report intragenomic unit length variants [5, 119], with some estimating that fewer than half of all units in an individual are of canonical size [26]. In the human T2T assembly, size differences appear to be chromosome‐specific and due to the number of R‐repeats downstream of the 3′ ETS [6]. These differences may not be obvious from conventional short‐read sequencing, so integrating long‐read data into rDNA variation analyses might prove essential.

## 4 Phenotypic Associations With rDNA Variation

**FIGURE 3:**
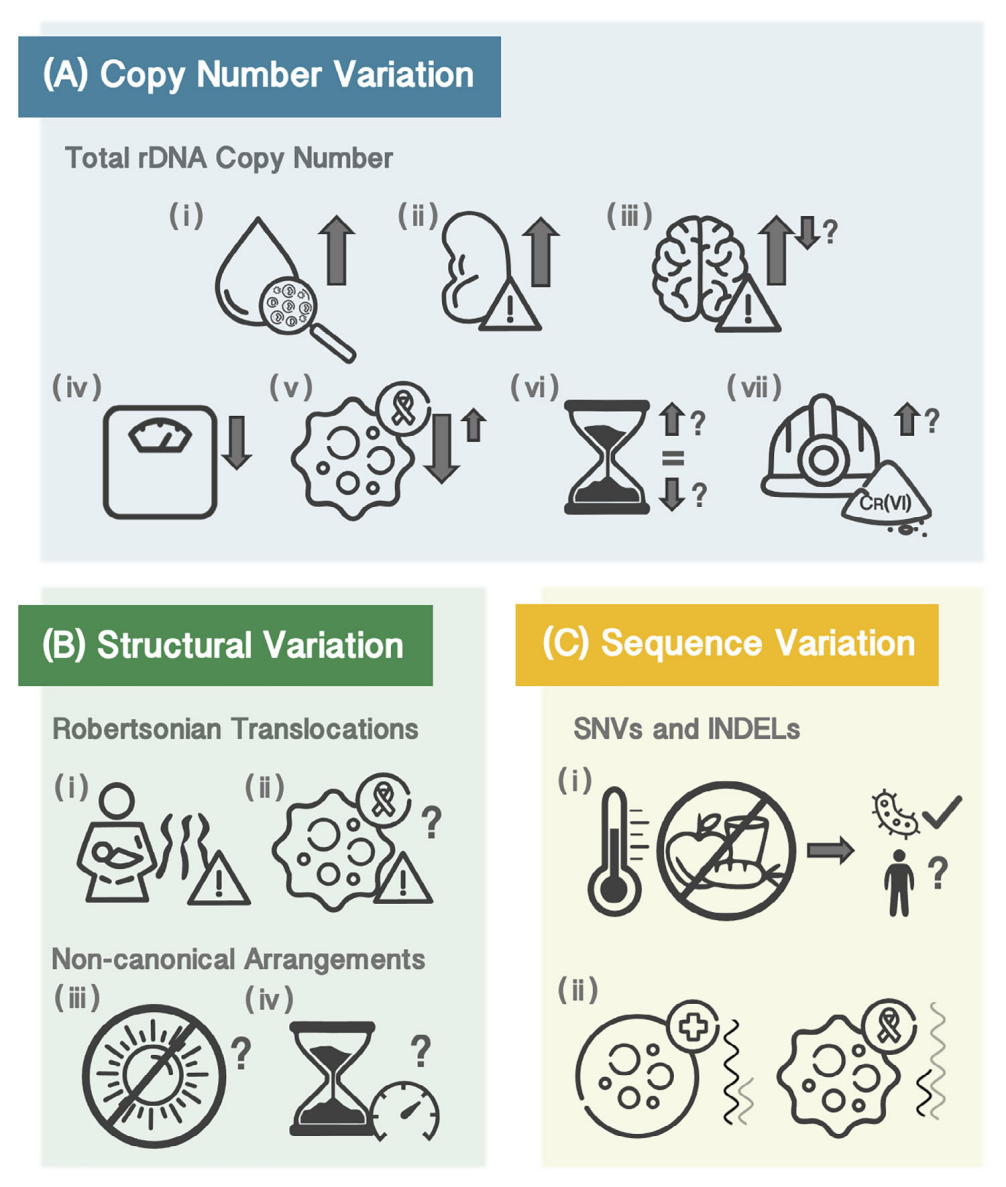
Putative phenotypic associations with rDNA variation. Schematic summary of associations between rDNA variants and human traits previously reported in the literature. (A) Higher rDNA CN has been associated with (i) blood cell subtype composition profiles akin to systemic inflammation, (ii) worse renal function and risk of kidney disease, as well as (iii) a variety of neuropsychological conditions, with some rDNA CN reduction reported in specific cases. Lower rDNA CN has also been reported to be associated with (iv) an increase in weight gain in adulthood. rDNA CN may also change under insults. Multiple studies observe (v) rDNA CN alterations in cancer. (vi) Loss of rDNA copies with age has been reported in many species, including humans, but recent studies contradict these findings. (vii) Exposure to Cr (VI) is reported to lead to rDNA CN increases, but results appear unreliable. (B) Regarding structural variation, Robertsonian Translocations lead to (i) a higher risk of offspring carrying chromosomal trisomies, and (ii) might increase the risk of cancer. Enrichment in non‐canonical rDNA unit arrangements has been reported in patients with (iii) Bloom syndrome—depicted here by their characteristic photosensitivity—and (iv) Werner syndrome—depicted here by their characteristic accelerated aging—, but recent studies question the extent of these associations. (C) Finally, (i) the presence of sequence variants appears to confer resilience to environmental stresses in lower organisms, but this has not been confirmed in humans. (ii) Some rDNA sequence variants appear to be expressed differentially between cancer and normal cells.

The widespread inter‐ and intra‐genomic variation in the human rDNA raises the obvious question of what, if any, are its consequences (Figure 3). Multiple studies have attempted to answer this, particularly with regard to CN differences. However, the increasing availability of high‐quality Biobank‐scale WGS cohorts with vast phenotypic records is only now starting to bring clarity.

### 4.1 Associations With Copy Number Variation

Given how fundamental to life protein synthesis is and the essential role that rRNA plays in ribosome biogenesis, it is no surprise that severe loss of rDNA copies leads to dramatic effects. Some animal species, such as frogs [168] and nematodes [74], can reach early larval stages without rDNA clusters, but die as soon as they exhaust their maternally‐inherited ribosomes. Partial reductions of rDNA CN are also known to cause the classic “bobbed” phenotype in flies [169], severe developmental impairments in nematodes [170], and increases in replication defects and sensitivity to DNA mutagens in yeast [171]. It is thus natural to assume rDNA CN variation affects ribosome biogenesis, particularly through changes in rRNA expression, but this may not necessarily be the case. A large portion of the rDNA copies in a cell are transcriptionally silent, as seen in human cells [172] and other species [173, 174]; differences in rDNA CN do not lead to concomitant changes in rRNA levels [172, 175]; and even artificially silencing copies fails to reduce rRNA expression, since the remaining active units increase their yield [38]. In both humans and mice, the proportion of silenced rDNA units changes in concordance with the total number of copies [5, 7, 175 ^–^ 177], suggesting some mechanism exists to keep the number of active copies tightly controlled. On the other hand, ribosome biogenesis levels appear to correlate with rDNA CN in skeletal muscle [178, 179], so perhaps the molecular mechanism at play acts separately from rRNA transcription. Various studies have reported changes in gene expression elsewhere in the genome associated with rDNA CN differences, particularly in those related to chromatin state and mitochondrial activity [71, 72, 180, 181]. This may arise from contacts between rDNA clusters and the rest of the genome in 3D space [182, 183], but further work remains to properly establish the underlying mechanism.

Regardless of the mechanism, rDNA CN has been implicated in a variety of human phenotypes (Figure 3A). For example, a recent study found an inverse correlation between rDNA CN and adult weight gain both in humans and rats [175]. A wide body of research has also focused on rDNA CN associations with neuropsychological disorders [184]. In particular, an elevated number of rDNA copies was observed in individuals with schizophrenia [70, 185], dementia with Lewy bodies [186], and intellectual disability [187], but elderly individuals with mild cognitive impairment appeared to harbor fewer copies than controls [188]. Higher rDNA CN was also found in UK Biobank (UKB) participants recorded as suffering from “other mental disorders,” but the most significant associations in that study related to blood cell composition and renal function [4]. Individuals with a relatively high number of rDNA copies had higher counts of neutrophils and/or platelets compared with lymphocytes, a pattern often interpreted as indicative of systemic inflammation, and lower glomerular filtration rate, indicative of worse kidney function, leading to a higher risk of kidney failure. On the other hand, the same study did not find any association between rDNA CN and age, despite the longstanding belief that the number of rDNA copies decreases with age [189]. This phenomenon has been observed in various organisms, particularly in yeast [61, 190] and flies [191], and would be consistent with rDNA loss through increased locus instability [192, 193]. Nevertheless, the evidence in mammals to date is largely contradictory, with several studies indeed reporting lower CN in older individuals [194 ^–^ 199], but other, more recent, studies reporting either no observable loss [4, 186, 200, 201] or even gain in some tissues [202]. A systematic longitudinal study following human participants throughout their lifespan would help settle these inconsistencies.

The instability of rDNA clusters makes them potentially susceptible to environmental insults. In this regard, a series of studies suggest exposure to Hexavalent Chromium (Cr (VI)) induces changes in rDNA CN [203 ^–^ 205]. These studies, however, report somewhat implausible effect sizes and contradictory results between in vivo and in vitro experiments. Methodological concerns thus question these observations. That being said, Cr (VI) is a known carcinogen, and it is widely accepted that cancer dramatically affects rDNA [206], similar to the CN alterations elsewhere in the genome that are a hallmark of cancer [207]. Most studies report losses of rDNA CN in tumor samples [77, 208, 209], although some identify both CN losses and gains depending on tumor type [210, 211]. These CN alterations are likely consequences of the widespread instability and not necessarily predictors. Nevertheless, smokers with higher rDNA CN measured prior to diagnosis appear to develop lung cancer at higher rates than those with low CN [212]. Whether this could extend to other cancer types and replicate in larger cohorts remains to be seen.

### 4.2 Associations With Structural Variation

To date, not much is known about whether rDNA structural variation has any phenotypic consequence beyond the context of Robertsonian translocations (Figure 3B). ROB carriers are widely thought to be phenotypically neutral, but with an elevated risk of siring offspring with Down's or Patau's syndromes due to their altered karyotype [111]. Analysis of almost 2000 ROB carriers, however, suggests these individuals might suffer from a higher risk of various cancers [213]. Whether this is a consequence of the loss of rDNA clusters remains unknown, since the impact of the number and size of particular clusters has not been explored yet in the general population, but evidence in other species suggests it may be the case [214]. Noncanonical cluster arrangements were initially estimated to make up approximately half of the rDNA units in Werner syndrome patients [119], but later analyses using long‐read sequencing observe only around 1.2% in that same condition and less than 2.5% in Bloom syndrome patients [5].

### 4.3 Associations With Sequence Variation

Variation within the nucleotide sequence of the rDNA units might also be associated with phenotypic outcomes (Figure 3C). For decades, it has been widely speculated that distinct rRNA sequences lead to ribosomes with specialized functionality [215], particularly as regulators of protein synthesis levels [156, 216 ^–^ 220], which may prove relevant at different developmental stages [221]. It is even possible not only that the efficiency at which particular mRNAs are translated depends on what variants are incorporated into ribosomes, but that distinct amino acid chains can arise from the same mRNA [222]. Other authors, however, have advised caution in interpreting any result as proof of ribosome specialization [223]. To date, the only studies that have convincingly shown this phenomenon happening were conducted in bacteria. In particular, in E. coli, ribosomes containing naturally‐occurring rRNA variants appear to regulate the expression of stress response genes [123]. Similarly, in *Vibrio vulnificus*, ribosomes harboring a specific rRNA variant preferentially translate a subset of mRNAs believed to permit these organisms to better adapt to changes in temperature and nutrient availability [224]. These observations suggest intragenomic rDNA variants selected against homogenization may enable better adaptability under environmental stress, which would be consistent with the high levels of rDNA sequence variation identified in microorganisms adapted to survive in extreme conditions [122, 225, 226].

Although no equivalent results exist yet in mammals, studies in mice show that rDNA subtypes are differentially silenced, with the level of silencing changing under nutritional stress [7, 227]. Naturally silenced variants can then get expressed when methylation is removed, as observed both in mouse [7] and human [9] cells, which could lead to the upregulation of specific rDNA variants under certain circumstances, such as after physical effort [228]. Recent evidence also suggests that the uneven silencing of rDNA variants in humans might be parent‐specific [9], not unlike the phenomenon of nucleolar dominance documented in multiple species [229, 230], paving the way to transgenerational epigenetic inheritance associated with rDNA sequence variation.

Much of the evidence supporting the phenotypic relevance of rDNA sequence variants in eukaryotes comes from differential rRNA expression of such variants in diverse scenarios [231]. Different tissues and cell types, for instance, show distinct rRNA transcription profiles in a wide variety of species, from plants [232] to mice [132] and humans [2, 8, 233, 234]. Some speculate that these differences might arise from distinct cell types prioritizing different properties of the ribosomes, such as translational fidelity and speed, that rRNA variants might facilitate [8]. Aside from rDNA CN changes, situations of increased DNA instability are prone to either the proliferation of rDNA sequence mutations or the overexpression of preexisting low‐abundance variants. This appears to be the case in senescence [202] and cancer [77, 146], with samples of distinct cancer types showing elevated expression of different rRNA variants [8].

Whether these changes in expression reflect changes in the underlying rDNA sequence and/or their regulation is still unclear. Nevertheless, a combination of both simultaneously appears likely, given that the rDNA CN alterations documented above are accompanied by changes in rDNA methylation [235], which we know is variant‐specific [7, 9], and some studies indicate different methylation states affect the likelihood of any given unit being lost [201]. Overall, this would suggest a scenario in which low abundance, and potentially detrimental, rDNA variants are kept silenced throughout most of an individual's lifespan. With aging or under another stressor, however, methylation may become destabilized and DNA breaks unsuitably repaired, triggering the overexpression of those previously suppressed variants. Even when all alleles are expressed during an individual's lifespan, subtle changes in the methylation levels of each allele (e.g., with aging) might lead to an imbalance in their expression. This could then lead to improper ribosome biogenesis and/or translational profile in the cell, and promote entering an oncogenic state. To date, however, no analyses at sufficient scale have been conducted that can associate the abundance of rDNA sequence variants with the risk of disease and establish whether such variants lead to distinct translational profiles in humans. These will all be essential to uncover the mechanisms linking rDNA variation with phenotype.

## 5 Uncovering the Mechanisms Driving the Consequences of rDNA Variation

Mounting evidence demonstrates that rDNA variation impacts human traits, but the molecular mechanisms underlying those influences remain obscure. Although it has long been speculated that rRNA variants may lead to ribosomes with distinct translational profiles [156, 215], evidence for it in mammalian cells is still lacking. To this end, mRNA engagement with polysomes measured with Polysome‐Seq [236] on a panel of diverse human cell lines may reveal which variants, and to what extent, affect protein synthesis in actively‐translating ribosomes.

Nevertheless, the regulation of protein translation may not be the only mechanism through which rDNA variation impacts human traits. Targeted RNase H‐mediated extraction of crosslinked RNA‐binding proteins (TREX) [237], for instance, can be applied to the spacer regions of pre‐rRNA transcripts to determine if variants within those regions affect binding of factors related to pre‐rRNA processing and ribosome biogenesis. Mass‐spectrometry‐based quantitative proteomics [238] can fulfill a similar role at the DNA level. Another route through which variants might impact traits is through changes in the rRNA modification landscape [239, 240], thus RNA modification estimates from long‐read single‐molecule data can reveal variant‐specific effects.

rDNA CN, on the other hand, may influence phenotype through changes in three‐dimensional chromosomal organization [241, 242]. The design of targeted rDNA‐capture high‐resolution Hi‐C panels [243] and its application to human genomes with a wide range of naturally‐occurring copy numbers can then reveal the extent to which such variation alters contacts and gene expression in finer detail than in the past [182]. Moreover, recent advances in genome editing in yeast suggest artificial reduction or increase of rDNA CN might be feasible [244], opening the door to comparisons within the same genetic background.

## 6 Conclusion

Research in the last few decades has shown that the extent of inter‐ and intra‐genomic variation in the human rDNA far exceeds prior expectations and that these variants influence multiple traits. It is now widely accepted that the rDNA clusters are hotspots for genomic instability and recombination, but, at the same time, the conservation of intragenomic variants across individuals and species must have been selected and thus confer evolutionary advantages. The availability of Biobank‐scale phenotype‐rich cohorts around the world will reveal the extent of the phenotypic impact of the diverse range of variant types and help settle contradictory past results. Moreover, the increasing interest in the field has already motivated the development of analysis methods explicitly targeted to the analysis of rDNA variation and its consequences. Nevertheless, much work still remains to fully comprehend the molecular mechanisms that link rDNA variation with human traits. Recent experimental advances promise to help provide clarity in this challenging, but exhilarating, prospect.

## Author Contributions

**Francisco Rodriguez‐Algarra**, **Vardhman K. Rakyan**: conceptualization. **Francisco Rodriguez‐Algarra**: literature review, writing. **Vardhman K. Rakyan**, **Elliott Whittaker**, **Sandra del Castillo del Rio**: editing and proofreading.

## Conflicts of Interest

The authors declare no conflicts of interest.

## Data Availability

Data sharing not applicable to this article as no new datasets were generated or analyzed.
